# Environmental Temperature, Other Climatic Variables, and Cardiometabolic Profile in Acute Myocardial Infarction

**DOI:** 10.3390/jcm13072098

**Published:** 2024-04-03

**Authors:** Cristina Vassalle, Daniele Grifoni, Bernardo Gozzini, Alessandra Parlanti, Luca Fibbi, Federica Marchi, Gianni Messeri, Nataliya Pylypiv, Alessandro Messeri, Umberto Paradossi, Sergio Berti

**Affiliations:** 1 Department of Laboratory Medicine, Fondazione Toscana Gabriele Monasterio, 56124 Pisa, Italy; 2 Laboratory of Monitoring and Environmental Modelling for the Sustainable Development (LaMMA Consortium), Via Madonna del Piano 10, 50019 Sesto Fiorentino, Italy; grifoni@lamma.toscana.it (D.G.); gozzini@lamma.toscana.it (B.G.); fibbi@lamma.toscana.it (L.F.); messeri@lamma.toscana.it (G.M.); 3 Institute of Bioeconomy (IBE), National Research Council (CNR), Via Madonna del Piano 10, 50019 Sesto Fiorentino, Italy; alessandro.messeri@ibe.cnr.it; 4 Diagnostic and Interventional Cardiology Department, Fondazione Toscana Gabriele Monasterio, Ospedale Pasquinucci, 54100 Massa, Italy; alessandra.parlanti@ftgm.it (A.P.); federica.marchi@ftgm.it (F.M.); pylypivn@ftgm.it (N.P.); umberto.paradossi@ftgm.it (U.P.); berti@ftgm.it (S.B.)

**Keywords:** acute myocardial infarction, STEMI, cardiovascular risk factors, prognosis, mortality, outside temperature, heat index, humidity, wind velocity, climate

## Abstract

**Objectives:** To evaluate CV profiles, periprocedural complications, and in-hospital mortality in acute myocardial infarction (AMI) according to climate. **Methods:** Data from 2478 AMI patients (1779 men; mean age 67 ∓ 13 years; Pasquinucci Hospital ICU, Massa, Italy; 2007–2018) were retrospectively analyzed according to climate (LAMMA Consortium; Firenze, Italy) by using three approaches as follows: (1) annual warm (May–October) and cold (November–April) periods; (2) warm and cold extremes of the two periods; and (3) warm and cold extremes for each month of the two periods. **Results:** All approaches highlighted a higher percentage of AMI hospitalization for patients with adverse CV profiles in relation to low temperatures, or higher periprocedural complications and in-hospital deaths. In warmer times of the cold periods, there were fewer admissions of dyslipidemic patients. During warm periods, progressive heat anomalies were characterized by more smoker (approaches 2 and 3) and young AMI patient (approach 3) admissions, whereas cooler times (approach 3) evidenced a reduced hospitalization of diabetic and dyslipidemic patients. No significant effects were observed for the heat index and light circulation. **Conclusions:** Although largely overlapping, different approaches identify patient subgroups with different CV risk factors at higher AMI admission risk and adverse short-term outcomes. These data retain potential implications regarding pathophysiological mechanisms of AMI and its prevention.

## 1. Introduction

The impact of temperature variations on human health has generated great interest, including impacts with regard to ST elevation myocardial infarction (STEMI), in which every decrease in a degree of temperature during the winter corresponds to an increase in hospital admissions [1,2,3].

Indeed, global warming has considerably altered many extreme climate events with notable mortality, economic, and environmental impacts. In particular, the frequency and severity of heat extremes have increased over most of the Earth, while those of cold extremes have shown decreasing trends. However, it should be kept in mind that these trends are not globally uniform and that cold extremes often occur suddenly, generating strong impacts [4]. To better evaluate all the possible impacts of climate change, it would therefore be advantageous not to evaluate the hot and cold extremes separately but jointly to better consider sudden changes in temperature. In the context of global climate change, extreme environmental temperatures will therefore be encountered more frequently, placing vulnerable individuals at increased risk [5]. Other meteorological variables, such as air humidity and wind, might modulate the relationship between temperature and cardiovascular health [6]. Identifying the most vulnerable subjects and implementing preventive measures can assume growing importance in view of global climate change and extreme events, even at our latitudes. In fact, the identification of the groups of patients at elevated risk will allow the implementation of measures to prevent the occurrence of acute events more targeted to the patient’s peculiarities.

The main objective of the present manuscript was to evaluate the risk profile in STEMI admissions according to the seasonal trend, outside temperature variation (with particular focus on extreme temperature), air humidity, and wind; periprocedural complications and in-hospital mortality were also assessed in relation to climate parameters. For this purpose, three approaches (annual warm and cold periods; warm and cold extreme days of the two periods; warm and cold extreme days for each month of the two periods) were used to consider the climate change impact on acute ischemic events.

## 2. Materials and Methods

### 2.1. Geographical Characteristics 

**Figure 1:**
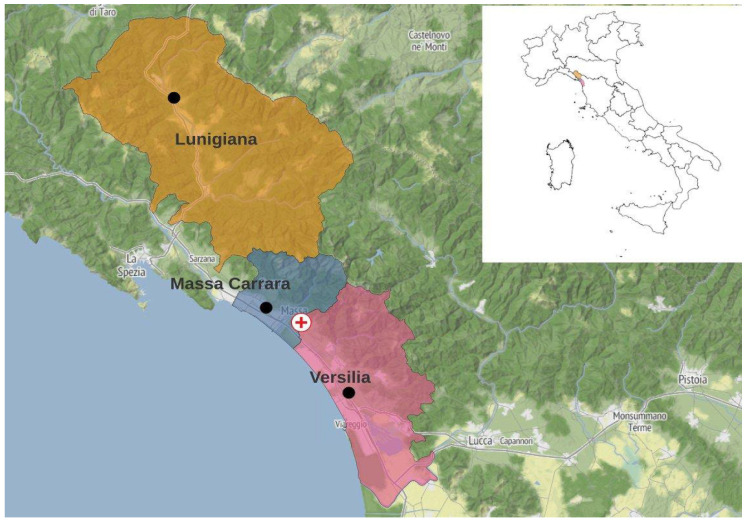
The areas of Lunigiana (orange), Massa Carrara (blue), and Versilia (purple) in Northwestern Tuscany. Meteorological data of each area were extracted on the black points, while the red cross indicates Pasquinucci Hospital in Massa. The area is characterized by a very complex orography due to the presence of the Apuan Alps and Apennine chain, while flat areas are present on the coast. This area was considered as the hospital catchment area.

The studied area (Northwestern Tuscany, Italy) can be classified in three subareas, including Lunigiana, Massa Carrara, and Versilia (Figure 1).

### 2.2. Meteorological Data

The LaMMA Consortium is an environmental monitoring and modeling laboratory that was founded by the Tuscany region in 1997 in cooperation with the Italian National Research Council (CNR) with the aim of creating an interface between the world of institutions, the components of scientific and technological excellence, industry, and various operational structures involved in the research and application of meteorology, and environmental modeling.

A database of meteorological parameters spatialized over the Tuscany region is available at LaMMA Consortium. The lack in the studied area of meteorological weather stations with long and complete data timeseries made it necessary the use of this type of data.

The daily outdoor spatialized data were calculated using the meteorological observations available in the Tuscany Region (Regione Toscana Servizio Idrologico Regionale, Aeronautica Militare, LaMMA Consortium, UCEA) archived in the LaMMA database for the period of 1991–2022. Weather station data, including minimum and maximum air temperature (Tmin and Tmax), relative air humidity (RH), mean sea level pressure (mslp), and wind speed, have been spatialized over Tuscany to obtain complete meteorological information from a spacetime point of view.

Spatialization was performed using an improved version of the Daymet algorithm [7], which generates a spatial interpolation of the meteorological observed variables using a digital terrain model (DTM). The original algorithm has been calibrated for the Tuscany region using a DTM with a spatial resolution of 250 m (in the area between 44.5719–42.1323 N latitude and 9.68646–12.474 W longitude). For this specific work, all the abovementioned meteorological spatialized data were extracted on a daily basis from one point of each specific subarea for the period of 2007–2018. Each point was selected in the most inhabited portion of the area. The coordinates of the extraction points for each subarea are as follows: Lunigiana Lat 44.352287 Lon 9.905089; Massa Carrara Lat 44.041161 Lon 10.084990; and Versilia Lat 43.914667 Lon 10.246351 (Figure 1).

Using air temperature and relative air humidity, we also calculated the heat index [8], also known as the apparent temperature or what the temperature feels like to the human body when relative humidity is combined with the air temperature. Specifically, in hot conditions, this variable may provide important indications for human body discomfort. There are specific values that identify risk thresholds (from no alert below 26.6 °C to extreme danger above 51 °C), and in this work, we used the threshold of 32.2 °C, beyond which starts the “extreme attention” class to calculate the monthly number of extreme days.

We also defined the days with atmospheric conditions favorable to the pollutant accumulation as the days characterized by mean sea level pressure higher than the 75° percentile and wind lower than the 25° percentile.

Three different approaches were used to verify the impact of meteorological conditions on acute ischemic events.

#### 2.2.1. Approach 1—Classification of Annual Warm and Cold Periods

To verify the presence of seasonality in the correlation between meteorological parameters and the occurrence of the pathologies studied in this work, the year was divided into cold and warm six-month periods (November/April and May/October, respectively). The warm and the cold periods were classified taking into account the air temperature averaged over the whole of the three areas; the six warmest and coldest months were therefore grouped together. The occurrence of acute ischemic events in the warm period was compared with that of the cold period.

#### 2.2.2. Approach 2—Classification of the Warm and Cold Extreme Days of the Two Periods

Using daily meteorological data (2007–2018), the 25° and 75° percentiles were calculated for the entire cold and warm periods for the three geographical areas (Supplementary Tables S1–S6).

This approach, which features the percentile calculated over a six-month period, allows us to identify days with the extreme meteorological conditions for each period in accordance with the chosen percentile. The percentiles, which were calculated for the two periods in each of the three subareas and for all meteorological variables, except for the heat index, were used to calculate the number of extreme days at a monthly level.

The comparison was performed with the other months of the period (when we analyzed the cooler months of the warm period and the mildest of the cold period to take into account the benefit of less extreme weather conditions) or of the remaining part of the year (when we analyzed the warmest of the warm period and the coldest months of the cold period).

#### 2.2.3. Approach 3—Classification of the Warm and Cold Extreme Days for Each Month of the Two Periods

Using daily meteorological data (2007–2018), the 25° and 75° percentiles were calculated for each month of the cold and warm periods for the three geographical areas (Supplementary Tables S7–S12). This approach allows us to identify days with extreme meteorological conditions for each month of the period with respect to its own climatology.

The occurrence of acute ischemic events in the months characterized by at least seven extreme days (in the cold or warm period) was then compared with that of the other months or of the remaining part of the year. The comparison was made as specified for approach 2.

### 2.3. Study Population Characteristics

Our Hub and Spoke network for STEMI (defined as in ref. [9]) in Northwestern Tuscany (population: 400,000, area: 1658 km^2^) began in April 2006 with the systematic use of percutaneous coronary intervention (PCI angioplasty with stent), involving one hub (Ospedale del Cuore di Massa) and five spoke centers, one medical helicopter, and six advanced life support ambulances, with direct transmission of prehospital ECG to the catheterization laboratory (24 h/7 days PCI capability within 30 min of notification) activated by a single-call action.

Patient information (e.g., demographic data, laboratory parameters, cardiovascular history, and risk factors) was extracted from the hospital’s computerized database. The extracted information including hypertension (blood pressure >140/90 mmHg/current use of antihypertensive drugs), dyslipidemia (DYS: lipid-lowering treatments/fasting low-density lipoprotein levels >150 mg/dL), type 2 diabetes (T2D; use of antidiabetic treatment/finding of HbA1c > 6.49%), smoking history (current/former smoking habit), and left ventricular function at admission (echocardiographic ejection fraction (EF) calculation). All patients presenting within 12 h of the onset of symptoms suggestive of myocardial ischemia and new or presumed new ST elevation or left bundle-branch block and treated with primary PCI (1/2007–12/2018) were considered for inclusion in this study. Due to the retrospective nature of the study and completed anonymization of patient data, which were analyzed as aggregates, the need to obtain informed consent was waived by the ethic committee.

### 2.4. Statistical Analysis

Continuous variables were reported as mean ± SD, and categorical variables were reported as number (percentage). Statistical analyses were performed using Student’s *t*-test, chi-square analysis, ANOVA and Scheffè test, Spearman test, and logistic univariate analysis; a *p* value < 0.05 was chosen as the level of significance.

## 3. Results

### 3.1. Meteorological Variables

#### 3.1.1. Approach 1—Classification of Annual Warm and Cold Periods

**Figure 2:**
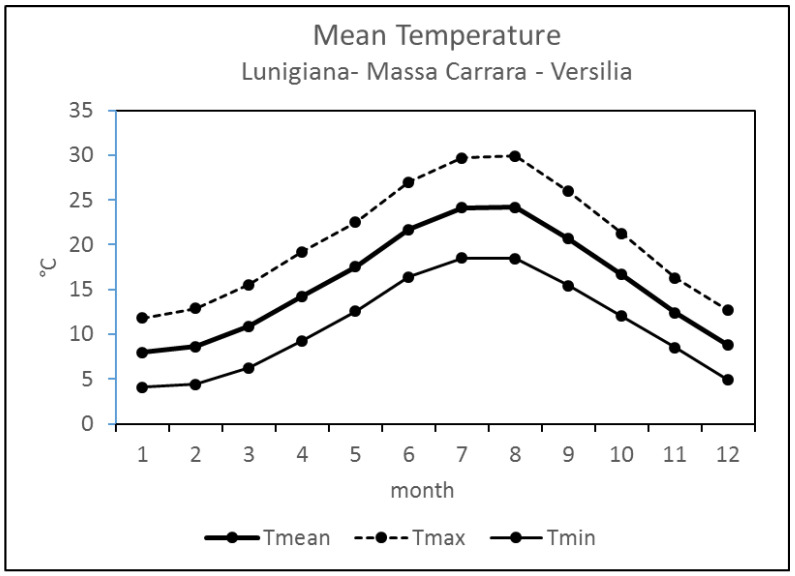
Monthly minimum (Tmin), maximum (Tmax), and mean (Tmean) temperature averaged over the Lunigiana, Massa Carrara, and Versilia areas, during the period of 2007–2018.

In Figure 2, the climatology of the minimum, maximum, and mean air temperature (2007–2022) for the whole study area is shown. On the basis of the climatological analysis, the year was classified in two subperiods of six months, including warm (from May to October) and cold (from November to April).

Considering the study period, which took place from January 2007 to December 2018, the mean daily temperature was 10.5 ± 3.3 °C in the cold period and 20.7 ± 3.6 °C in the warm period.

The Spearman correlation coefficient was calculated between monthly meteorological parameters for warm and cold periods (Table 1).

The results highlight a strong expected positive correlation between Tmin, Tmax, and Tmean both for warm and cold periods. In the warm period, a significant negative correlation between relative humidity (RH) and Tmin, Tmax, and Tmean is evident; a similar negative correlation, although there was a lower level of significance, was also observed in the cold period, but only for Tmax and Tmean.

High levels of RH are generally associated with mild and humid westerly cloudy flux, which reduces the nighttime cooling and the incident solar radiation during the day, especially in the warm period. Wind speed was negatively correlated with Tmin in both periods and with Tmean in the cold period, while mslp was negatively correlated with wind speed and only in the warm period with RH. These correlations could be explained by the passage of weather fronts, which determine a falling of mslp and an increase in wind speed and on the other hand, lead to an increase of RH and a falling of minimum air temperature.

#### 3.1.2. Approach 2—Calculation of 25° and 75° Percentiles for the Warm and Cold Periods (2007–2018)—Classification of the Warm and Cold Extreme Days of the Two Warm and Cold Periods

Calculated 25° and 75° percentiles for the entire warm and cold periods (2007–2018) were categorized as follows:

- Days with meteorological parameters above the 75° percentile. For air temperature, days warmer (warm period) or less cold (cold period) than normal;
- Days with meteorological parameters between the 75° and 25° percentile; days without extremes;
- Days with meteorological parameters lower than the 25° percentile; days less warm (warm period) or colder (cold period) than normal. In Supplementary Tables S1–S6, the seasonal percentiles calculated for warm and cold periods are shown.

#### 3.1.3. Approach 3—Calculation of 25° and 75° Percentiles for the Single Month of the Warm and Cold Period (2007–2018)—Classification of the Warm and Cold Extreme Days for Each Month of the Two Warm and Cold Periods

In Supplementary Tables S7–S12, the monthly percentiles calculated for each month of the warm and cold periods are shown.

### 3.2. Impact of Meteorological Variables on STEMI Patient Characteristics

#### 3.2.1. Characteristics of STEMI Patients in the Overall Population and after Stratification According to the Classification of Annual Warm (May–October) and Cold (November–April) Periods (Approach 1)

A total of 2478 STEMI patients was evaluated (1779 men; male/female ratio: 2.5; mean age 67 ∓ 13 yrs). AMI patients hospitalized in the cold period (November/April) were elderly and more often T2D and DYS and presented with periprocedural cardiovascular complications more often compared to those hospitalized in the warmer period (May/October) (Table 2).

#### 3.2.2. Characteristics of STEMI Patients Stratified According to the Warm and Cold Extreme Days of the Warm and Cold Periods (Approach 2)

##### Cold Period Extremes

Months in the cold period had 7 or more days of minimum temperatures below the 25° percentile of the cold period and/or with maximum temperatures below the 25° percentile of the cold period (colder than normal) compared with the remaining months of the year over the period of 2007–2018.

More T2D (24 vs. 20%, χ^2^ = 5.5, *p* < 0.05) and DYS (49 vs. 37%, χ^2^ = 18, *p* < 0.001) patients were admitted. A higher number of periprocedural cardiovascular complications were also registered (3.9 vs. 1.9%, χ^2^ = 6.6, *p* < 0.01), as well as a higher number of in-hospital deaths (5.1 vs. 3%, χ^2^ = 5.4, *p* < 0.05).

Months in the cold period had 7 or more days with minimum temperatures higher than the 75° percentile of the cold period and/or with maximum temperatures higher than the 75° percentile of the cold period (warmer than normal) compared with the other months in the cold period (2007–2018)

A lower number of DYS patient were admitted (39 vs. 49%, χ^2^ = 7.9 *p* < 0.01).

##### Warm Period Extremes

Months in the warm period had 7 or more days with maximum temperatures higher than the 75° percentile of the warm period (warmer than normal) compared with the remaining months of the year (2007–2018)

More smokers (44 vs. 39%, χ^2^ = 3.8, *p* ≤ 0.05) were admitted.

Months in the warm period had 7 or more days with minimum temperatures lower than the 25° percentile of the warm period and/or with maximum temperatures lower than the 25° percentile of the warm period (cooler than normal) compared with the other months in the warm period (2007–2018).

There were no significant effects on the demographic and clinical characteristics.

#### 3.2.3. Characteristics of STEMI Patients Stratified According to the Classification of the Warm and Cold Extreme Days for Each Month of the Two Warm and Cold Periods (Approach 3)

##### Monthly Extremes of the Cold Period

Months in the cold period had 7 or more days with minimum temperatures below the 25° percentile of the month and/or with maximum temperatures below the 25° percentile of the month (colder than normal) compared with the remaining months of the year (2007–2018).

More T2D (24 vs. 20%, χ^2^ = 6.2, *p* ≤ 0.01), DYS (46 vs. 37%, χ^2^ = 12.5, *p* < 0.001) and elderly (68 ± 13 vs. 67 ∓ 13, *p* < 0.05) patients were admitted. A higher number of in-hospital deaths were also registered (4.8 vs. 3%, χ^2^ = 4.8, *p* < 0.05). 

Months in the cold period had 7 or more days with minimum temperatures higher than the 75° percentile of the month and/or with maximum temperatures higher than the 75° percentile of the month (warmer than normal) compared with the other months in the colder period (2007–2018).

Fewer DYS patients were admitted (41 vs. 49%, χ^2^ = 5.1, *p* < 0.05).

##### Monthly Extremes of the Warm Period

Months in the warm period had 7 or more days with maximum temperatures higher than the 75° percentile of the month (warmer than normal) compared with the remaining months of the year (2007–2018).

**Figure 3:**
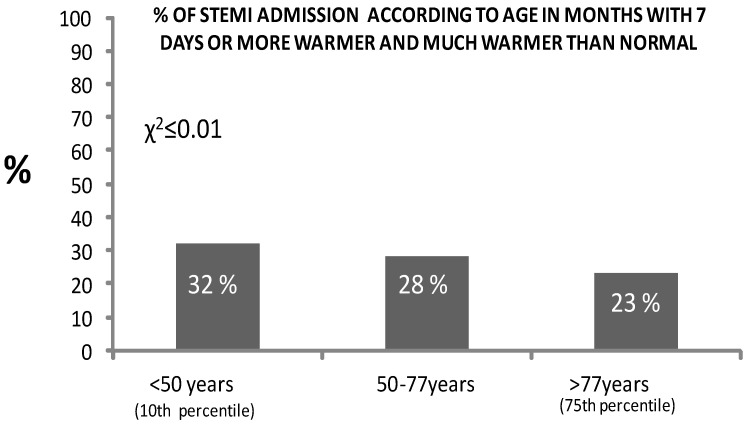
Percentage of STEMI patients stratified for age classes according to period with 7 days/month or more with maximum temperatures higher than the 75° percentile of the month in question (May–October, warmer and much warmer days than normal).

More smokers (44 vs. 40%, χ^2^ = 6, *p* ≤ 0.01) and younger patients (66 ± 13 vs. 68 ± 13, *p* < 0.05) were admitted. In particular, more admissions of patients < 50 years of age were observed (Figure 3).

Months in the warm period had 7 or more days with minimum temperatures lower than the 25° percentile of the month and/or with maximum temperatures lower than the 25° percentile of the month (cooler than normal) compared with the other months in the warm period (2007–2018)

There were fewer admissions of T2D (17 vs. 22%, χ^2^ = 4.2, *p* < 0.05) and DYS patients (34 vs. 44%, χ^2^ = 9, *p* < 0.01).

### 3.3. Characteristics of STEMI Patients Stratified According to the Heat Index (>32.4 °C)

There were no significant effects on the demographic and clinical parameters.

### 3.4. Months with 7 Days or More with Light Circulation

There were no significant effects on the demographic and clinical parameters.

### 3.5. Logistic Regression Analysis

The risk of being hospitalized for AMI in patients presenting specific CV risk factors according to outdoor temperature conditions is reported in Table 3, Table 4 and Table 5 (approaches 1–3, respectively).

## 4. Discussion

Our results highlight that sensitivity to temperature anomalies varies in STEMI patients with different CV risk factors and profiles, also affecting short-term outcomes. Cold appeared more harmful than heat. In addition, although largely overlapping, the use of the different approaches (considering mean periods, period extremes or monthly extremes for the cold and warm periods) may identify patient subgroups with different CV risk profiles and short-term outcomes at higher risk of being hospitalized for an acute ischemic event.

Many gaps remain in identifying determinants that may lead to plaque rupture, as traditional risk factors only partially explain variations in AMI incidence. Indeed, seasonal variation in AMI has been hypothesized before the first half of 1900 [10,11], and after these first observations, a seasonal variation in the occurrence of AMI (winter peak) has been confirmed for many years and in many geographical territories, especially in areas with cold and temperate climate [12,13]. Rather than using the conventional division into seasons, we have divided the year into two periods based on temperatures in a more targeted way. Overall, we registered a slight increase in the number of STEMI cases in warm weather rather than in the cold period; however, it must be considered that the Ospedale del Cuore covers a touristic area, which is prone to marked fluctuations due to seasonal movements in the population number. In consideration of this fact, as STEMI incidence must be considered in view of the number of individuals present in the area (unfortunately impossible to evaluate precisely, but that surely even doubles/triples during summer holidays), admissions in our center are likely in line with previous results reporting a greater AMI rate in winter. Really, we indirectly confirmed the closer relationship between the cold seasons and AMI incidence, as our data showed an association between low outdoor temperature and a greater percentage of AMI patients with specific cardiovascular risk profiles (T2D, dyslipidemia, older subjects), and as such, are potentially more vulnerable to environmental triggers. Possible underlying mechanisms are numerous (e.g., blood pressure, heart rate, myocardial oxygen consumption, glucose and lipid profile, hematological and coagulation factor as fibrinogen, platelets, body weight, inflammatory markers as IL-6 and C-reactive protein, many clustering with higher level trends in the colder months) [14,15]. Moreover, cold air breathing may reduce the immune response capacity and induce pulmonary neurogenic reflexes and consequently increase the risk of arrhythmias and atherothrombosis, especially in patients with preexisting respiratory disease. In addition, changes in lifestyle habits (e.g., increase in alcohol consumption, change in physical activity and dietary habits) in the cold period have been proposed as acute ischemic event trigger [13]. Also, common respiratory viral infections, which showed a peak related to low temperature, must be taken into account [16]. Clearly, all these factors may exacerbate the basal condition in more vulnerable patients and increase the risk of acute event precipitation. Accordingly, when we performed a first analysis with approach 1, differences in patient characteristics and clinical factors according to the annual warm and cold periods was evidenced; overall, STEMI patients admitted in the cold period were more often males, T2D, and dyslipidemic and more frequently experienced periprocedural complications. Other results confirmed that the cold period shows increased the risks for AMI, especially for the patients with T2D or DYS [12,17]. In this context, fasting glucose and HbA1c levels are found to be significantly higher in colder than in warmer periods [18,19]. More specifically, a greater frequency of admission for AMI in T2D with respect to non-T2D patients has been previously found, especially in the extreme temperature range of both cold and hot periods [20]. Moreover, many studies support the link between seasonal differences in lipid levels, with elevated values in winter, as well as a negative association evidenced between environmental air temperature and lipids [21,22]. Among underlying mechanisms that may be responsible for this trend, hemoconcentration, as well as seasonal changes in the liver’s production of cholesterol and the activity of lipid receptors and lipases, have been identified as possible factors, although other variables (e.g., variations in physical activity, diet and other lifestyle-related parameters) may contribute for the observed changes in lipid profile [23,24,25]. It is also known that patients with T2D have impaired endothelial function and poor skin blood flow, which imply impaired thermoregulation, especially in response to extreme temperature fluctuations, together with numerous other mechanisms, including inflammation/oxidative stress, sweating, and dehydration, which characterize these patients [14]. It is also known that insulin can directly modulate the hypothalamic neurons that regulate thermogenesis, accelerate metabolism, and act on brown fat and is therefore involved in the process of regulating body temperature [26]. Interestingly, a role for vitamin D, which is synthesized by the skin following exposure to ultraviolet radiation and shows a strong seasonal dependence, has been suggested for this relationship, as patients with T2D have reduced levels of 25(OH)D [27,28]. Accordingly, in a subgroup of AMI patients (n = 123), in which vitamin D tests were available, levels of 25(OH)D were lower in T2D compared with non-T2D patients (mean:14 ± 8 vs. 22 ± 11 mg/dL, *p* ≤ 0.001), and the percentage of patients with 25(OH)D marked severe (< 10 mg/dL) was more than tripled in T2D compared with non-T2D patients (48% vs. 13%, *p* < 0.001). Moreover, other data suggested that the odds of having insulin resistance was significantly greater for subjects in the lowest quartile of 25(OH)D compared to the other quartiles combined, likely by modulation of several key pathways affecting glucose metabolism (e.g., increasing proinflammatory cytokines and oxidative stress affecting insulin signaling and epigenetic regulation of gene expression, as well as RAAS) [28,29,30,31]. It has been also suggested that low vitamin D is associated with atherogenic blood lipid profiles, whereas its supplementation is able to lower the levels of total cholesterol, triglycerides, and LDL cholesterol, as well as to increase HDL cholesterol [32]. Notably, as AMI patients showed a marked percentage of patients with hypovitaminosis D, the role of vitamin D in T2D, dyslipidemia, and AMI with annual solar radiation changes merits further investigation in future studies [33].

When we applied approaches 2 and 3, evidencing period and month weather extremes, other risk factors emerged. In particular, a higher admission rate of smokers in hot periods, which could be related to indirect effects, such as an increase in dyspnea, especially in patients with breathing-related problems (e.g., chronic obstructive pulmonary disease) [34]. In addition, the coronary vasoconstrictive effects of tobacco smoking could facilitate the onset of acute events, especially in more vulnerable subjects, such as those with previous a myocardial infarction or a history of a revascularization procedure [35].

Interestingly, although summertime is generally considered as a recreational time of the year, we also found an increased admission of young AMI patients (<50 years) associated with heat in the warm period when using approach 2. AMI occurred less frequently in young adults aged <50 years; accordingly, the young group accounted for less than 10% of all cases in the present study. Previous data on Italian subjects living in Florence also confirmed this trend related to hot conditions [36]. One possible reason is that younger people can be more exposed to hot extreme temperatures because of working outdoors and other physical activities. In our population, younger AMI patients were mostly male (86%) and presented with different characteristics from those over the age of 50 years in terms of CV risk factors. A smoking habit represented an important risk factor, accounting for almost two-thirds of young AMI patients (77%); however, young AMI patients are less likely to present with T2D (6%), hypertension (35%), and DYS (34%). However, more frequent exposure to cigarette smoking can result in endothelial dysfunction and increased risk of CVD, especially when starting at an early age [37]. Also, dyslipidemia appears to be one of the most common risk factors in young AMI patients. Interestingly, HDL, which was available in a subset of patients, was lower in the young AMI group than it was in older patients (42 ± 13 vs. 46 ± 13, respectively *p* < 0.001). Another possible additive explanation is the presence of psychosocial stressors, which are associated with an increased risk of acute myocardial infarction in general but are particularly important in younger AMI subjects, as exposure to high stress is associated with the risk of myocardial infarction in young adults [38,39]. Previous data have evidenced that high stress, together with vigorous physical activity in untrained subjects, may trigger acute cardiac events within a few hours after arrival to the tourist destination, as well as by occasional heavy physical exertion, suggesting that this young patient population might incur these kinds of external triggers [40,41].

As it concerns outcomes, we observed that patients who suffered AMI in the cold period more often presented with in-hospital cardiovascular periprocedural complications and mortality compared to those hospitalized in the warm period (approaches 1 and 2). It has been previously observed that low temperatures in colder periods increase the odds of overall mortality, and in particular, cold stress was identified as a major risk factor for mortality [42,43,44]. Moreover, extremely low temperatures in winter appeared to be associated with an increased risk of AMI in different studies [45,46,47,48]. Indeed, the increased risk of periprocedural complication and in-hospital mortality in colder periods may be due to thermal stress, the expression of exposure to extreme (cold or hot) temperatures, as previously suggested [49,50]. Interestingly, previous data reported that the effects of temperature lowering toward AMI were stronger in years with higher average temperatures and during summer, suggesting that an unusual temperature anomaly might be more relevant than a temperature decrease in absolute values in terms of health effects [2,51].

An interesting aspect in this field is the fact that body temperature is an indicator of physiological functions; accordingly, some studies have evidenced that moderate hypothermia may protect tissues from acute injury [52]. In particular, the idea of therapeutic hypothermia has been proposed and tested in patients with various diseases, also showing potential beneficial therapeutic effects on AMI in view of the promising results obtained in experimental studies and early clinical trials [52]. In this context, the potential of mild temperature hardening in patients with cardiovascular disease to increase their resistance to climatic fluctuations and to reduce the risk of the AMI (as a sort of ischemic preconditioning in which short periods of ischemia render the myocardium more resistant to following ischemic damage [53]) and the signaling pathways by which this might be achieved, may open a difficult but exciting area of study focusing on the relationship between temperature and AMI.

We failed to observe any significant relationship for other meteorological variables, such as humidity and wind speed. Previous available results were controversial for these and other meteorological variables (e.g., atmospheric pressure and wind) [54,55], although some reasonable mechanisms may indeed support an effect (e.g., humidity may alter the processes of sweat and body temperature homeostasis, increasing the respiratory fatigue and heart rate).

## 5. Study Strengths and Limitations

The study is retrospective and reflects only a single-center experience. The value of the meteorological parameters used in this study must be considered as an average value to which the population has been subjected, as individual outdoor exposure to weather conditions is impossible to collect. Other variables, such as indoor conditions or seasonal behaviors (e.g., diet, activity, psychosocial factors), may affect exposure to outdoor temperature.

Advantages of our study include multidisciplinary collaborations between clinicians and meteorologists, accurate meteorological assessment, the use of large datasets, and more than 10 years of AMI hospitalization and mortality data. We additionally considered light circulation, which indirectly provides an estimation of air pollution. The majority of available studies focused on the standard deviation of temperatures but did not discriminate between temperatures that were higher or lower than the average (e.g., period or monthly extremes). Moreover, previous stratified analyses were generally performed for all AMI patients, without considering whether temperature effects vary by different subgroups with preexisting disease or risk factors (e.g., T2D or DYS).

## 6. Conclusions

Identifying the most vulnerable subjects at higher risk for AMI can assume growing importance in view of global climate change and extreme events, even at our latitudes, thereby allowing for the implementation of preventive behavioral changes more targeted to the patient’s peculiarities (educational planning: e.g., adequate clothing and diet, control of time spent outdoors, adequate domestic heating/air conditioning, etc., as well as the development of tools: e.g., temperature-dependent health warning system for groups of patients with specific risk profile).

## Figures and Tables

**Table 1:** Spearman correlation coefficient (*p* value) between monthly meteorological variables of all areas during the warm and cold periods.

Warm Period					
	Tmax	Tmean	RH	Wind	Mslp
Tmin	**0.85**	**0.95**	**−0.56**	−0.15	−0.05
Tmax		**0.97**	**−0.52**	−0.04	−0.05
Tmean			**−0.56**	−0.09	−0.05
RH				**0.16**	**−0.24**
Wind					**−0.55**
Cold period					
	Tmax	Tmean	RH	Wind	Mslp
Tmin	**0.86**	**0.96**	0.06	**−0.21**	−0.10
Tmax		**0.97**	**−0.30**	−0.08	−0.03
Tmean			**−0.20**	−0.14	−0.07
RH				−0.03	−0.09
Wind					**−0.38**

Bold indicates a significance level <0.01, while underlined indicates a significance level <0.05.

**Table 2:** Demographic and clinical characteristics in the overall studied population and according to annual warm and cold periods.

Parameters	Overall Population (n = 2478)	Warm Period (May/October) (n = 1312)	Cold Period (November/April) (n = 1166)	*p* Value
Age (years)	67 ∓ 13	67 ∓ 13	68 ∓ 13	≤0.05
Age (>77 yrs–75th percentile)	616 (25)	312 (24)	304 (26)	ns
Age (<50 yrs–10th percentile)	227 (9)	126 (10)	101 (9)	ns
Males	1779 (72)	937 (71)	842 (72)	ns
Diabetes	515 (21)	248 (19)	267 (23)	≤0.01
Hypertension	1418 (57)	738 (56)	680 (58)	ns
Dyslipidemia	740 (30)	371 (28)	369 (31)	≤0.01
Current smokers	981 (40)	544 (41)	437 (37)	ns
Creatinine (mg/dL, at admission)	1.2 ∓ 0.8	1.2 ∓ 0.8	1.2 ∓ 0.7	ns
CAD familiarity	628 (25)	341 (26)	287 (25)	ns
Previous MI, PCI/CABG	337 (14)	171 (13)	166 (14)	ns
Left ventricular ejection fraction-LVEF (at admission) (%)	44 ∓ 10	44 ∓ 10	43 ∓ 10	ns
LVEF < 40%	312 (13)	154 (12)	158 (13)	ns
Periprocedural cardiovascular complications	60 (2.4)	23 (1.7)	37 (3.2)	<0.05
In-hospital mortality	89 (3.6)	39 (3.0)	50 (4.3)	0.08

ns = not significant.

**Table 3:** Risk for patients with specific CV risk factors to be hospitalized for AMI and incur in periprocedural complications and adverse outcomes according to outdoor conditions (approach 1).

	Approach 1—November/April vs. May/October
Parameters	OR (95% CI) *p*
Age (one year)	1.006 (1–1.01) ≤ 0.05
Type 2 diabetes	1.28 (1.1–1.6) ≤ 0.01
Dyslipidemia	1.27 (1.1–1.5) ≤ 0.01
Periprocedural complications	1.8 (1.1–3.1) < 0.05

**Table 4:** Risk for patients with specific CV risk factors to be hospitalized for AMI and incur in periprocedural complications and adverse outcomes according to outdoor conditions (approach 2).

Approach 2—Period Extremes (November/April vs. May/October)			
Parameters	Coldest Extremes of the Cold Period (Tmin < 25th Percentile and/or Tmax < 25th Percentile)	Warmer Extremes of the Cold Period (≥ 7 days Tmin > 75th Percentile and/or Tmax > 75th Percentile)	Warmest Extremes of the Warm Period (≥7 days Tmax > 75th Percentile)
	OR (95% CI) *p*		
Type 2 diabetes	1.3 (1.1–1.6) < 0.05	-	-
Dyslipidemia	1.6 (1.3–1.6) < 0.001	0.7 (0.5–0.9) < 0.01	-
Smokers	-	-	1.2 (1–1.5) ≤ 0.05
Periprocedural complications	2.0 (1.2–3.4) < 0.01	-	-
In-hospital mortality	1.7 (1.1–2.7) < 0.05	-	-

**Table 5:** Risk for patients with specific CV risk factors to be hospitalized for AMI and incur in periprocedural complications and adverse outcomes according to outdoor conditions (approach 3).

Approach 3—Monthly Extremes				
Parameters	Coldest Extremes of the Cold Period (Tmin < 25th Percentile and/or Tmax < 25th Percentile)	Warmer Extremes of the Cold Period (≥7 Days Tmin > 75th Percentile and/or Tmax > 75th Percentile)	Warmest Extremes of the Warm Period (≥7 Days Tmax > 75th Percentile)	Coldest Extremes of the Warm Period (≥7 Days Tmin < 25th Percentile)
	OR (95% CI) *p*			
Age (one year)	1.008 (1.001–1.002) *p* < 0.05		0.98 (0.98–0.99) < 0.001	
Type 2 diabetes	1.3 (1.1–1.6) ≤ 0.01	-	-	0.7 (0.6–1) < 0.05
Dyslipidemia	1.4 (1.2–1.7) < 0.001	0.7 (0.5–1) < 0.05	-	0.7 (0.5–0.9) < 0.01
Smokers	-	-	1.2 (1–1.5) ≤ 0.01	
Periprocedural complications	-	-	-	
In-hospital mortality	1.6 (1.1–2.5) < 0.05	-	-	

## Data Availability

Data will be made available on request for research purposes to those responsible for the meteorological and clinical databases.

## Supplementary Materials

The following supporting information can be downloaded at: https://www.mdpi.com/article/10.3390/jcm13072098/s1.
