# Supplementary material for: Environmental Temperature, Other Climatic Variables, and Cardiometabolic Profile in Acute Myocardial Infarction
Source: J Clin Med. 2024 Apr 3;13(7):2098. doi: 10.3390/jcm13072098 (PMC11012411; doi:10.3390/jcm13072098)
Supplement: Supplementary file 1 [file jcm-13-02098-s001.zip › jcm-2929820-supplementary.pdf]

Supplementary Table S1. Percentile values for the whole warm period for Lunigiana

perc	Tmin (°C)	Tmax (°C)	Tmean (°C)	RH (%)	Wind (m/s)	Mslp (hPa)
0.75	16.1	29.8	22.7	15.0	81.4	1.8
0.25	13.8	26.1	19.9	12.9	74.7	1.5

Supplementary Table S2. Percentile values for the whole cold period for Lunigiana

perc	Tmin (°C)	Tmax (°C)	Tmean (°C)	RH (%)	Wind (m/s)	Mslp (hPa)
0.75	7.5	16.3	11.7	12.4	87.6	2.3
0.25	4.4	13.4	9.0	9.0	78.7	1.6

Supplementary Table S3. Percentile values for the whole warm period for Versilia

perc	Tmin (°C)	Tmax (°C)	Tmean (°C)	RH (%)	Wind (m/s)	Mslp (hPa)
0.75	19.2	29.1	24.2	10.9	75.8	1.7
0.25	16.9	26.2	21.5	9.6	70.4	1.3

Supplementary Table S4. Percentile values for the whole cold period for Versilia

perc	Tmin (°C)	Tmax (°C)	Tmean (°C)	RH (%)	Wind (m/s)	Mslp (hPa)
0.75	9.8	17.3	13.4	10.2	81.6	1.8
0.25	7.0	15.0	11.1	8.4	75.5	1.3

Supplementary Table S5. Percentile values for the warm period for Massa Carrara

perc	Tmin (°C)	Tmax (°C)	Tmean (°C)	RH (%)	Wind (m/s)	Mslp (hPa)
0.75	19.5	29.6	24.5	11.1	74.7	1.7
0.25	17.2	26.7	21.9	9.8	68.8	1.4

Supplementary Table S6. Percentile values for the cold period for Massa Carrara

perc	Tmin (°C)	Tmax (°C)	Tmean (°C)	RH (%)	Wind (m/s)	Mslp (hPa)
0.75	9.9	17.6	13.6	10.1	79.7	1.9
0.25	7.3	15.3	11.4	8.4	73.1	1.4

Supplementary Table S7. Percentile values for each month of the whole warm period for Lunigiana

	perc.	Tmin (°C)	Tmax (°C)	Tmean (°C)	RH (%)	Wind (m/s)	Mslp (hPa)
May	0.75	12.5	24.8	18.2	81.6	1.9	1017.0
	0.25	9.0	19.6	14.7	66.0	1.3	1008.5
June	0.75	15.9	29.6	22.4	79.6	1.7	1017.0
	0.25	12.9	24.4	18.9	68.4	1.3	1008.8
July	0.75	17.8	31.8	24.6	76.9	1.7	1016.4
	0.25	14.7	28.2	21.6	65.8	1.3	1006.4
Aug	0.75	17.6	32.1	24.7	78.4	1.7	1016.3
	0.25	14.8	27.9	21.7	61.6	1.2	1001.7
Sep	0.75	15.5	28.0	21.4	82.8	1.9	1017.4
	0.25	11.5	23.4	17.7	68.1	1.3	1004.2
Oct	0.75	12.7	22.6	17.4	88.4	2.0	1019.8
	0.25	7.5	18.0	13.1	73.6	1.1	1007.7

Supplementary Table S8. Percentile values for each month of the whole cold period for Lunigiana

	perc.	Tmin (°C)	Tmax (°C)	Tmean (°C)	RH (%)	Wind (m/s)	Mslp (hPa)
Nov	0.75	9.7	17.1	13.1	90.2	2.3	1020.2
	0.25	4.1	12.9	8.7	76.0	1.2	1005.0
Dec	0.75	5.6	13.2	9.2	92.4	2.1	1025.2
	0.25	0.2	9.4	5.3	71.4	1.1	1008.1
Jan	0.75	7.1	14.0	10.3	83.6	1.7	1022.4

	0.25	2.5	11.0	7.0	68.5	0.9	1008.0
Feb	0.75	7.4	15.0	11.0	81.4	1.8	1021.6
	0.25	3.0	11.9	8.0	64.9	0.9	1006.5
Mar	0.75	8.6	17.3	12.7	78.6	1.9	1020.4
	0.25	5.2	14.3	10.0	59.4	1.0	1004.7
Apr	0.75	11.6	21.0	15.9	77.0	1.8	1017.2
	0.25	8.5	17.2	13.1	61.7	1.0	1004.1

Supplementary Table S9. Percentile values for each month of the whole warm period for Versilia

	perc.	Tmin (°C)	Tmax (°C)	Tmean (°C)	RH (%)	Wind (m/s)	Mslp (hPa)
May	0.75	14.8	24.3	19.3	77.1	1.7	1016.8
	0.25	11.8	20.4	16.2	64.4	1.1	1007.7
June	0.75	18.7	28.9	23.8	76.4	1.7	1016.9
	0.25	15.9	24.7	20.3	64.5	1.1	1008.6
July	0.75	20.9	30.9	25.8	72.8	1.7	1016.5
	0.25	18.3	28.1	23.3	63.1	1.1	998.6
Aug	0.75	20.7	30.9	25.7	72.5	1.7	1016.3
	0.25	18.3	28.1	23.3	61.4	1.0	994.0
Sep	0.75	18.1	27.7	22.9	76.0	1.7	1017.6
	0.25	14.4	24.3	19.7	63.5	1.0	1003.1
Oct	0.75	15.0	23.4	19.1	80.2	1.6	1020.1
	0.25	10.5	19.9	15.5	69.2	0.9	1006.0

Supplementary Table S10. Percentile values for each month of the whole cold period for Versilia

	perc.	Tmin (°C)	Tmax (°C)	Tmean (°C)	RH (%)	Wind (m/s)	Mslp (hPa)
Nov	0.75	12.0	18.9	15.4	82.2	1.9	1020.3
	0.25	6.8	15.4	11.0	70.6	0.9	1004.1
Dec	0.75	8.4	15.0	11.6	84.4	1.8	1025.0
	0.25	3.0	11.9	7.7	70.1	0.9	1008.2

Jan	0.75	7.1	14.0	10.3	83.6	1.7	1022.4
	0.25	2.5	11.0	7.0	68.5	0.9	1008.0
Feb	0.75	7.4	15.0	11.0	81.4	1.8	1021.6
	0.25	3.0	11.9	8.0	64.9	0.9	1006.5
Mar	0.75	8.6	17.3	12.7	78.6	1.9	1020.4
	0.25	5.2	14.3	10.0	59.4	1.0	1004.7
Apr	0.75	11.6	21.0	15.9	77.0	1.8	1017.2
	0.25	8.5	17.2	13.1	61.7	1.0	1004.1

Supplementary Table S11. Percentile values for each month of the warm period for Massa Carrara

	perc.	Tmin (°C)	Tmax (°C)	Tmean (°C)	RH (%)	Wind (m/s)	Mslp (hPa)
May	0.75	15.2	24.7	19.7	75.8	1.7	1017.0
	0.25	12.2	20.9	16.6	60.3	1.2	1007.9
June	0.75	19.0	29.1	24.0	75.1	1.7	1016.9
	0.25	16.2	25.2	20.6	62.6	1.2	1008.6
July	0.75	21.2	31.4	26.2	71.5	1.7	1016.4
	0.25	18.7	28.4	23.7	61.0	1.2	1003.3
Aug	0.75	21.0	31.4	26.1	71.3	1.7	1016.3
	0.25	18.7	28.4	23.7	58.7	1.2	997.9
Sep	0.75	18.5	28.3	23.4	74.6	1.7	1017.4
	0.25	15.1	24.9	20.2	60.9	1.2	1002.9
Oct	0.75	15.3	23.9	19.5	78.9	1.7	1020.0
	0.25	10.9	20.3	15.8	66.1	1.1	1006.6

Supplementary Table S12. Percentile values for each month of the whole cold period for Massa Carrara

	perc.	Tmin (°C)	Tmax (°C)	Tmean (°C)	RH (%)	Wind (m/s)	Mslp (hPa)
Nov	0.75	12.3	19.2	15.5	80.8	1.9	1020.3
	0.25	7.3	15.8	11.6	68.1	1.0	1004.5
Dec	0.75	8.6	15.3	11.9	82.6	1.7	1025.0
	0.25	3.6	12.1	8.1	66.2	1.0	1008.0
Jan	0.75	7.2	14.2	10.5	81.7	1.9	1022.3
	0.25	3.1	11.2	7.2	64.7	1.0	1008.1

Feb	0.75	7.4	15.3	11.2	79.4	1.9	1021.7
	0.25	3.4	12.2	8.2	61.1	1.1	1006.5
Mar	0.75	8.9	17.6	13.0	77.6	2.0	1020.4
	0.25	5.5	14.5	10.3	54.8	1.2	1004.9
Apr	0.75	11.9	21.4	16.3	75.8	1.9	1017.2
	0.25	8.8	17.5	13.4	57.7	1.2	1004.3
